# Co-occurring mental illness in emergency department and hospital inpatient encounters related to substance abuse in Maryland

**DOI:** 10.1186/1940-0640-10-S1-A22

**Published:** 2015-02-20

**Authors:** Ting-Ying Jane Huang, Jeanne Yang, Patience Moyo, Linda Simoni-Wastila

**Affiliations:** 1 Pharmaceutical Health Services Research, University of Maryland School of Pharmacy, Baltimore, MD, 21201, USA; 2 Pharmaceutical Research Computing, University of Maryland School of Pharmacy, Baltimore, MD, 21201, USA

## Background

Mental illness and substance abuse are highly comorbid conditions. At the individual level, symptoms of one can influence symptoms of the other and therefore demand treatment approaches specific to the combination of substance abused and mental illness. At the population level, co-occurring substance use and mental illness challenge public health prevention efforts and decrease treatment system effectiveness. However, due to the scarcity of integrated data, there has not been a thorough understanding of co-occurrence of substance use and mental illness. This study examines prevalence and trends of mental illness among substance abuse-related emergency department (ED) and hospital inpatient encounters.

## Methods

This descriptive study used data from the 2008 to 2012 Maryland Health Services Cost Review Commission (HSCRC), which contain medical record abstracts and billing information from inpatient admissions, ED visits, outpatient surgeries, and clinic visits from all Maryland hospitals. Because HSCRC data are reported at the encounter-level, they may include multiple encounters by the same patient. Only ED and hospital inpatient encounters were analyzed for this study. Encounters related to substance abuse (including alcohol, opioids, sedative-hypnotics, and other psychotropic agents) were identified using associated International Classification of Diseases, 9^th^ revision (ICD-9) codes (alcohol/drug dependence and abuse 303-305.93; alcoholic chronic conditions 357.5, 425.5, 535.3, 571.x, 790.3; alcohol/drug poisoning 965.x, 967-970.9, 980.x, E850-E860.0, E939.4, E950.xx, E962.0, E980.x) listed as any of the discharge diagnoses. Co-occurring mental illness was identified from the same record using ICD-9 mental disorder codes 290.xx-314.xx, except for those codes used to identify encounters related to substance abuse. We reported prevalence of co-occurring mental illness by year and admission type. Trend analyses were conducted by examining slope estimates from logistic regression modeling for proportions.

## Results

The total number of substance abuse-related ED and inpatient encounters increased from 128,941 in 2008 to 156,142 in 2012. ED encounters as a proportion of total encounters grew from 43 percent to 55 percent (p = 0.001) and started exceeding inpatient encounters in 2011. ED encounters were more likely younger (mean_ED_ 39 vs. mean_inpatient_ 45 years-old) and male (male_ED_ 65% vs. male_inpatient_ 62%), while inpatient encounters were more likely nonwhite (nonwhite_ED_ 44% vs. nonwhite_inpatient_ 47%). Prevalence of co-occurring mental illness among substance abuse-related encounters increased from 53 percent to 57 percent for ED (p < 0.001) and from 78 percent to 79 percent (p < 0.025) for inpatient encounters. The increasing trends were driven by the increasing co-occurring mental illness in alcohol abuse ED encounters and in alcohol and opioid abuse inpatient encounters.

## Conclusions

This analysis demonstrated the feasibility of using hospital encounter data to identify co-occurrence of substance use and mental illness, thereby improving prevention and intervention efforts. Both the proportions of substance abuse-related ED and inpatient encounters with co-occurring mental illness increased in recent years in Maryland. Findings suggested ED encounters are more likely younger, male, and white race. Future public health interventions should attend to the different population characteristics in substance abuse cases presenting to different settings when developing prevention strategies.

